# Supplementary material for: Applying Protein–Protein Interactions and Complex Networks to Identify Novel Genes in Retinitis Pigmentosa Pathogenesis
Source: Int J Mol Sci. 2022 Apr 2;23(7):3962. doi: 10.3390/ijms23073962 (PMC8999418; doi:10.3390/ijms23073962)
Supplement: Supplementary file 1 [file ijms-23-03962-s001.zip › ijms-1636340-supplementary.pdf]

SUPPLEMENTARY RESULTS

Table S1. Gene Ontology Enrichment Data on Biological Processs for Intermediate genes. The biological processes cover a collection of molecular activities that can be seen as a larger program.

#term ID	term description	observed gene count	background gene count	false discovery rate	matching proteins in your network (labels)
**GO:0021987**	cerebral cortex development	3	109	0.0273	CDH2,GART,RHOA
**GO:0060019**	radial glial cell differentiation	2	12	0.0273	CDH2,RHOA

Table S2. Gene Ontology Enrichment Data on Molecular Functions for Intermediate genes. This table lists GTPase activity as the only molecular activity carried out by the gene product in our network based on the known databases.

#term ID	term description	observed gene count	background gene count	false discovery rate	matching proteins in your network (labels)
**GO:0003924**	GTPase activity	3	283	0.0350	GNG2,GNGT1,RHOA

Table S3. Gene Ontology Enrichment Data on Cellular Components for Intermediate genes. The cellular components suggest localization of gene products according to the cell anatomy.

#term ID	term description	observed gene count	background gene count	false discovery rate	matching proteins in your network (labels)
**GO:0019897**	extrinsic component of plasma membrane	4	145	0.00013	CDH2,GNG2,GNGT1,RHOA
**GO:0031234**	extrinsic component of cytoplasmic side of plasma membrane	3	106	0.0013	GNG2,GNGT1,RHOA
**GO:0098552**	side of membrane	4	436	0.0023	GNG2,GNGT1,ITGB2,RHOA
**GO:0005834**	heterotrimeric G-protein complex	2	29	0.0030	GNG2,GNGT1
**GO:0098797**	plasma membrane protein complex	4	502	0.0030	CDH2,GNG2,GNGT1,ITGB2
**GO:0044432**	endoplasmic reticulum part	5	1294	0.0047	CDH2,DOLK,EVA1A,PNPT1,RHOA
**GO:0101003**	ficolin-1-rich granule membrane	2	61	0.0079	ITGB2,RHOA
**GO:0005789**	endoplasmic reticulum membrane	4	1022	0.0171	DOLK,EVA1A,PNPT1,RHOA
**GO:0044853**	plasma membrane raft	2	95	0.0171	CDH2,ITGB2
**GO:0098590**	plasma membrane region	4	1061	0.0171	CDH2,GNGT1,ITGB2,RHOA
**GO:0098827**	endoplasmic reticulum subcompartment	4	1025	0.0171	DOLK,EVA1A,PNPT1,RHOA
**GO:0031090**	organelle membrane	6	3337	0.0281	DOLK,EVA1A,GNGT1,ITGB2,PNPT1,RHOA
**GO:0030496**	midbody	2	165	0.0297	PLK1,RHOA
**GO:0044425**	membrane part	8	6517	0.0298	CDH2,DOLK,EVA1A,GNG2,GNGT1,ITGB2,PNPT1,RHOA
**GO:0030027**	lamellipodium	2	185	0.0326	CDH2,RHOA
**GO:0101002**	ficolin-1-rich granule	2	186	0.0326	ITGB2,RHOA
**GO:0098805**	whole membrane	4	1554	0.0438	CDH2,EVA1A,ITGB2,RHOA
**GO:0044459**	plasma membrane part	5	2651	0.0473	CDH2,GNG2,GNGT1,ITGB2,RHOA
